# Mechanistic insights into how cichoriin inhibits P2Y_14_R to suppress MSU-induced gouty inflammation

**DOI:** 10.1186/s12896-026-01151-z

**Published:** 2026-04-15

**Authors:** Xue Wu, Shanshan Liu, Li Leng, Zhengqi Liu

**Affiliations:** 1 Guizhou University of Traditional Chinese Medicine, Guiyang, Guizhou, 550025 China; 2 The Second Affiliated Hospital of Guizhou University of Traditional Chinese Medicine, Guiyang, Guizhou 550003 China

**Keywords:** Cichoriin, Gout, Inflammation, Pyroptosis, Molecular dynamics simulation

## Abstract

**Background:**

Gout is a prevalent, chronic inflammatory joint diseases, and its global prevalence and incidence are continue to rise. Currently, the adverse side effects of anti-gout drugs underscore the urgent need for safer and more effective anti-gout agents.

**Objective:**

Cichoriin is a kind of coumarin, which exhibits diversity of biological activities. The current investigation aimed to explore the mechanism of the inhibition of MSU-induced gouty inflammation by cichoriin.

**Methods:**

The enzyme inhibitory assay of P2Y_14_R, cell viability detection, ELISA, immunofluorescence staining, and flow cytometry were used to explore the molecular mechanism of cichoriin in the inhibition of MSU-induced gouty inflammation. The molecular level details of inhibitory effects of chchoriin against P2Y_14_R were obtained by molecular dynamics simulation.

**Results:**

The in vitro experiments revealed that cichoriin could inhibit the activity of P2Y_14_R, up-regulate the expressions of NLRP3, Caspase−1, GSDMD and ASC, increase IL−1β and IL−18 levels, and decrease the percentage of Caspase−1/PI double-positive cells. The computational calculations revealed that cichoriin and P2Y_14_R could form a stable and rigid complex. Free energy landscape exhibited that cichoriin stabilized the global conformations of P2Y_14_R to the minimum global energy. MM-PBSA provided evidence for cichoriin’s stability inside the binding pocket of P2Y_14_R with a binding free energy of -35.13 kcal/mol. The decomposition of binding energy showed the pivotal amino acids residues responsible for the stability of cichoriin’s interaction with P2Y_14_R by forming hydrogen bonds and hydrophobic interactions.

**Conclusions:**

This work highlighted the potential roles of cichoriin in attenuating MSU-induced gouty inflammation.

**Supplementary Information:**

The online version contains supplementary material available at 10.1186/s12896-026-01151-z.

## Introduction

Gout is regarded as a prototypical inflammatory disease. Gouty arthritis (GA) is the main clinical manifestation of gout. It is characterized by the deposition of monosodium urate (MSU) in articular cavity and other soft tissue space. The condition could trigger acute inflammatory responses, leading to intense pain, swelling, lingering discomfort, and limited joint motion, severely affecting the mood states, daily living, and social functioning in persons with GA [1, 2]. It has been recognized as one of the most common chronic inflammatory joint diseases, and its prevalence and incidence is steadily increasing worldwide due to the changed human diets and lifestyles [3, 4]. Patients with GA often experience asymptomatic periods between acute attacks, during which urate crystals may keep accumulating and causing damage. Over time, chronic gout can trigger joint deformity and irreversible joint destruction [5]. Currently, most of anti-gout drugs just alleviate symptoms or postpone disease onset and even have adverse side effects, so the search for safer and more efficacious drugs is a hot research topic [6].

Gout is caused by excessive deposition of MSU crystal in joints [7]. The formed MSU crystals could cause disordered purine nucleotide catabolism and activate Nod-like receptor family, pyrin domain containing 3 (NLRP3) inflammasome [8]. NLRP3 inflammasome is composed of a hierarchical architecture, with NLRP3, apoptosis-associated speck-like protein (ASC), and caspase-1, and closely associated with the course of GA [9, 10]. The activated NLRP3 inflammasome could trigger downstream signals including gasdermind D (GSDMD)-mediated pyroptotic cell death and the secretion of pro-inflammatory cytokines, which could effectuate diverse immune responses [11]. It has been reported that abnormal activity of NLRP3 and dysregulated expression of NLRP3 inflammasome members were observed in human GA patients and animal models [12, 13], and the factors leading to the activation of NLRP3 inflammasome are under intense focus currently [14].

Purine receptors are a kind of transmembrane receptor, and could govern immune response process and multiple physiological functions by selectively binding to extracellular nucleoside or nucleotide [15]. Purine receptors are generally divided into adenosine (P1) and nucleoside (P2) receptors. The latter can be further subdivided into ligand-gated ion channel receptors (P2X receptors, P2XR) and G protein-coupled receptors (P2YR) [16]. The G protein-coupled receptor P2Y_14_R could be stimulated by uridine diphosphate glucose (UDP-G) and uridine diphosphate glucose sugars (UDP-sugars), and it coupled to Gi/o protein to further trigger subsequent signal transduction pathway [17]. It has been reported that MSU could induce P2Y_14_R overexpression in human keratinocytes with a significantly increased release of inflammatory cytokines, implying the role of P2Y_14_R in MSU-triggered inflammatory responses [18]. In addition, the activation of P2Y_14_R also has close association with intracellular levels of cAMP, which negatively regulates the activation of NLRP3 inflammasome [19]. In the meantime another study reported that P2Y_14_R receptor plays essential roles in acute GA, and intracellular cAMP might participate in the regulation of P2Y_14_R in NLRP3 inflammasome-mediated acute GA [20]. P2Y_14_R probably regulates the inflammatory cascade through NLRP3 inflammasome via cAMP in acute GA [21]. Therefore, the modulation of P2Y_14_R activity may provide a potential therapeutic target for exploiting targeted agents for gout.

Coumarin represents a class of low-molecular weight phenolics, and its chemical structure is composed of fused benzene and α-pyrone rings [22]. Coumarins acting anti-inflammatory biological activities has been reported [23]. It is noteworthy that cichoriin (6-hydroxy-7-O-glucosylcoumarin or aesculetin 7-glucoside) as a member of coumarin displays several interesting bioactivities, including anti-inflammatory [24], antioxidant [25], and antibacterial [26]. Cichoriin is indicated to be isolated from plants, fungi, and bacteria and has an average synthetic accessibility [25, 27]. Coumarins showed the potential of anti-gout effects [28]. However, their influences to P2Y_14_R activity have not been characterized. In view of the above, our study is devoted to evaluate the regulation of cichoriin to gouty inflammation based on the inhibition effect of cichoriin on the activity of P2Y_14_R. In vitro experiments and in silico approaches were used to explore the molecular mechanism of cichoriin in suppressing MSU-induced gouty inflammation.

## Materials and methods

### Cell sources and culture conditions

The HEK293 cell line was purchased from Procell (Wuhan, China). The complete culture medium was prepared with 89% minimum essential medium (MEM), 10% fetal bovine serum (FBS), and 1% penicillin-streptomycin (P/S), and incubated in incubator at 37℃ with 5% CO₂. When the cell density reached approximately 80%, the cells were passaged at 1:2 ratio. The THP-1 cell line was obtained from Procell (Wuhan, China). These cells were cultured in a complete medium composed of 88.9% RPMI-1640, 10% FBS, 1% P/S, and 0.1% β-mercaptoethanol. The cells were maintained in incubator at 37℃ with 5% CO₂, and passaged at 1:3 ratio.

### P2Y_14_R inhibition activity assay

The HEK293 cell line stably expressing P2Y_14_R was cultured in complete medium at 37℃ with 5% CO₂. The cells were seeded at a density of 2 × 10⁵ cells/ml in 384-well plate with 50 µl per well. After 24 h, the wells were washed with sterile PBS. After that, 7.5 µl of induction buffer containing 500 µM IBMX (MCE, HY-12318), 100 µM Ro 20-1724 (MCE, HY-100927), 30 µM Forskolin (MCE, HY-15371), and 10 µM UDP-G (MCE, HY-N7032) was added, along with different concentrations of PPTN or cichoriin (0.01 nM, 0.1 nM, 1 nM, 10 nM, and 100 nM). The plates were incubated at 37℃ for 30 min, and compounds at each concentration were tested in triplicate. The intracellular cAMP levels were measured by using the cAMP-Glo™ Assay Kit (Promega, WI, USA).

### Cell viability assay

When THP-1 cell density exceeded 90%, cells were seeded in 96-well plate at a density of 3 × 10⁵ cells/ml and treated with 100 ng/ml phorbol 12-myristate 13-acetate (PMA) for 48 h to induce differentiation into macrophages. Subsequently, different concentrations of PPTN or cichoriin were added. After 24 h, 100 µl of medium containing 10% CCK8 reagent was added into each well followed by incubated at 37℃ in the dark for 2 h. The optical density at 450 nm was measured using a microplate reader with three replicates per group.

### Enzyme-linked immunosorbent assay (ELISA)

MSU was purchased from MCE, and was dissolved in sterile PBS to prepare a 50 mg/ml suspension. The suspension was solubilised by heating and sonication. After the differentiation of THP-1 monocytes into macrophages, the culture medium was removed, followed by washing twice with sterile PBS. Subsequently, the cells were stimulated with 250 µg/ml MSU suspension in complete medium to create an inflammatory environment.

THP-1 cells were divided into six groups, including blank group, model group, PPTN group, as well as high, medium, and low-dose groups, with three replicates per group. After induced with 100 ng/ml PMA for 48 h, cells were pre-treated with cichoriin or PPTN for 1 h, and then stimulated with 250 µg/ml MSU suspension for 23 h. The supernatants were collected for detecting the expression of inflammatory cytokines IL-1β and IL-18. The ELISA kit (neobioscience) was used according to the manufacturer’s instructions, and OD values were measured at 450 nm.

### Detection of NLRP3 expression by immunofluorescence

THP-1 cells were seeded at a density of 10⁶/ml in 6-well plates with cell culture inserts. After the differentiation of THP-1 monocytes into macrophages, the cells were pre-treated with PPTN or cichoriin for 1 h, followed by co-culture with 250 µg/ml MSU suspension for 23 h. The cells were fixed with 4% paraformaldehyde for 30 min at room temperature and then washed three times with PBS. After that, cells were permeabilized with 0.2% Triton X-100 in PBS for 30 min on ice and blocked with 5% BSA for 1 h at room temperature. The primary antibody anti-NLRP3 (Wanleibio, WL02635, 1:100) was added and incubated overnight at 4℃. After washing with PBS, the secondary antibody (1:100) was added and incubated for 60 min at room temperature in the dark. The cells were washed again with PBS and stained with DAPI for 5 min in the dark for nuclear counterstaining. After washing with PBS, the cell culture inserts were mounted on slides with anti-fade mounting medium. The samples were monitored by a fluorescence microscope. The mean fluorescence intensity of NLRP3 in each group was measured using ImageJ software.

### Western blot analysis

After 24 h of compound exposure, cells were lysed on ice for 30 min with RIPA buffer containing 1% protease and phosphatase inhibitors, followed by centrifugation at 12,000×g for 15 min at 4 °C. The supernatant was collected and quantified with bicinchoninic acid assay kit. Samples were mixed with 5×loading buffer to final concentration of 2 µg/µL, and then boiled at 100 °C for 10 min to ensure complete denaturation, followed by aliquoted and stored at − 80 °C.

Twenty µg of protein was loaded and separated by electrophoresis at 150 V constant voltage, and then transferred to membranes at 400 mA constant current. The membranes were blocked with 5% non-fat milk in 1×TBST for 1 h at room temperature on a shaker, followed by overnight incubation with primary antibodies against anti-NLRP3 (Abcam, ab263899), anti-Caspase-1 (Abcam, ab207802), anti-GSDMD (Abcam, ab210070), and anti-ASC (Abcam, ab151700) at 4 °C. The next day membranes were washed 5 times with 1×TBST at room temperature, followed by incubation with HRP-conjugated secondary antibody (PMK Biotechnology, Wuhan, 1:20000) for 1 h, and then washed again as above. Protein bands were visualized with ECL reagent, and band intensities were quantified by using ImageJ software.

### Cellular thermal shift assay (CETSA)

The 50 µM cichoriin was added to HEK293 cells. The control group was treated with an equivalent volume of DMSO. After incubated for 1 h, the cells in the two groups were collected. Cells were heated at a range of temperatures (48, 50, 52, 54, 56, and 58 ℃) for 10 min. Subsequently, the cells were frozen and thawed repeatedly for lysis. After that, the samples were centrifuged at 4 ℃ for 10 min. Different concentrations of cichoriin (0, 2, 4, 8, 16, 32, 64 µM) were incubated with HEK293 cells at 54 ℃ for 10 min for screening of concentrations. Western blot analysis was used for the detection.

### Flow-cytometric detection of pyroptosis

After induced with 100 ng/ml PMA for 48 h, THP-1 cells were exposed to compounds and detached with trypsin. Following centrifugation cells were resuspended in 1×wash buffer and detected by FLICA 660 Caspase-1 Assay kit (Immunochemistry Technologies) according to the manufacturer’s instructions. In brief, cells were mixed with 5 µL of 30× FLICA 660 reagent and 295 µL complete medium and incubated for 60 min at 37 °C in the dark. After that, cells were washed with 1×wash buffer, centrifuged at 3000 rpm for 5 min at room temperature, and resuspended in fresh wash buffer. After adding PI, flow cytometry was used to measure stained cells.

### Statistical analysis

Data were analyzed with R language. Quantitative results were expressed as mean ± standard deviation (SD). The univariate analyses of normally distributed data with homogeneity of variance were performed by analysis of variance (ANOVA), otherwise Kruskal-Wallis tests were applied. A *P*-value < 0.05 was considered statistically significant.

### Homology modelling

The amino acid sequence was retrieved from GenBank database. The sequence similarity search for P2Y_14_R was applied by using NCBI-BLAST search in Protein Data Bank (PDB). A crystal structure of P2Y12R (PDB ID: 4pxz) was used as template for building structure of P2Y_14_R. The homology model of P2Y_14_R was constructed by using Modeller version 10.2. The best conformation was selected by molpdf assessment score. The stereochemical quality of homology model was assessed by using PROCHECK and ERRAT programs available from SAVES server (https://saves.mbi.ucla.edu/), and Molprobity and QMEANDisco scores from SWISS structure assessment service(https://swissmodel.expasy.org/).

### Molecular docking

Molecular docking was performed by using Autodock 4.2.6 for analyzing the interaction between PPTN/cichoriin and P2Y_14_R. Polar hydrogens and Gasteiger charges were added to P2Y_14_R and ligand. A grid box was obtained with grid space of 60 × 60 × 60 for X, Y, and Z coordinates for P2Y_14_R. Lamarckian genetic algorithm was used for molecular docking. Autodock tools 1.5.6 was applied for post-docking analysis.

### Molecular dynamics (MD) simulation

MD simulation was applied to analyze the dynamical structural information of P2Y_14_R as well as P2Y_14_R-PPTN and P2Y_14_R-cichoriin complexes by using Gromacs 2025 package at a 100 nano seconds (ns) time scale. CHARMM36 all-atom force field was used to cichoriin and P2Y_14_R. TIP3P explicit water solvation model was applied in an orthorhombic periodic boundary box, and 10 Å distance was set between complex and box. In order to neutralize the charge of the system, Cl^−^ ions were added. After that, energy minimization for the solvated system was performed via 100,000 steps of steepest descent algorithm. Stabilization followed by sequential NVT(number of particles, volume, and temperature) and NPT (number of particles, pressure, and temperature) equilibration were required. The particle mesh ewald method was used to compute electrostatic interactions. A 200 picosecond (ps) NVT equilibration was performed at a 300 K temperature, and a 200 ps NPT equilibration was used to keep the system pressure at 1 bar. Finally, unrestrained 100 ns MD simulation was carried out with a timestep of 2 femtoseconds (fs), and a snapshot was obtained for every 10 ps. The Root mean square deviation (RMSD), root means square fluctuation (RMSF), radius of gyration (Rg), solvent accessible surface area (SASA), hydrogen bond, dynamic cross-correlation matrix (DCCM), free energy landscape (FEL), and molecular mechanics Poisson–Boltzmann surface area (MM-PBSA) analyses were conducted.

## Results

### Interaction between P2Y_14_R and cichoriin

**Fig. 1:**
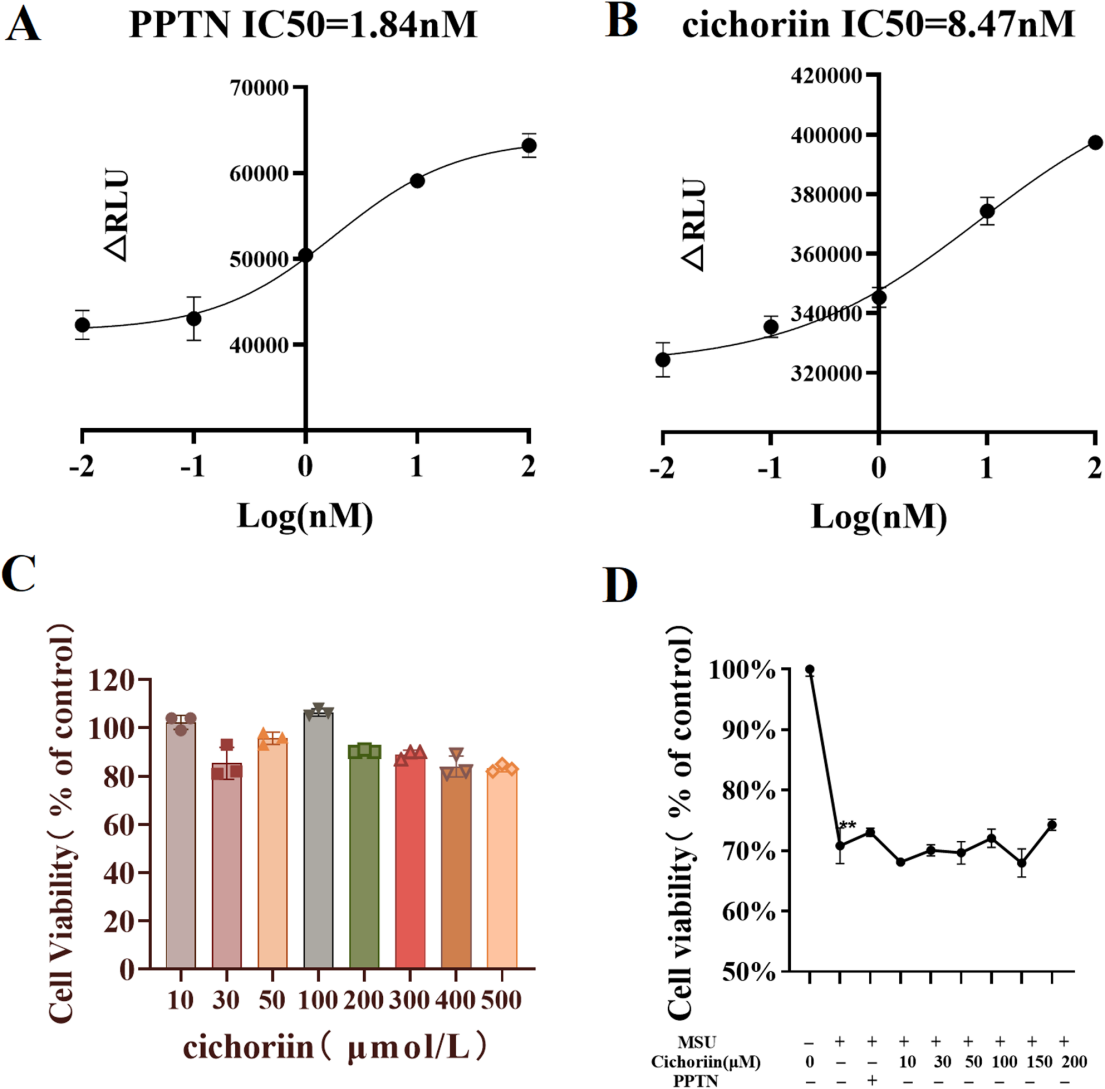
(**A**) The chemiluminescence curve for the inhibition of PPTN against P2Y_14_R; (**B**) The chemiluminescence curve for the inhibition of cichoriin against P2Y_14_R; (**C**) Impact of cichoriin on THP-1 macrophage viability; (**D**) Impact of cichoriin on MSU-stimulated macrophage activity

P2Y_14_R-inhibitory activity of cichoriin was evaluated by measuring cAMP accumulation in HEK293 cells which stably express human P2Y_14_R. PPTN, a well-characterized P2Y_14_R antagonist, was included as a positive control. The half maximal inhibitory concentration (IC50) value of PPTN is 1.84 nM for P2Y_14_R. Cichoriin demonstrated potential inhibitory activity against P2Y_14_R (IC50 = 8.47 nM). The concentration–response curve is depicted in Fig. 1A and B.

### Effect of cichoriin on THP-1 macrophage viability

THP-1-derived macrophages were exposed to increasing concentrations of cichoriin for assessing cytotoxicity. As shown in Fig. 1C, cichoriin at 10–500 µM did not alter cell viability compared with control group. MSU significantly reduced cell viability (*P* < 0.01). Neither PPTN (10 µM) nor cichoriin (10–200 µM) reversed this decline (*P* > 0.05) compared with MSU group. Nevertheless, modest upward trends were observed at 50, 100 and 200 µM of cichoriin (Fig. 1D). Consequently, the three concentrations were selected as the low, medium, and high doses for subsequent experiments.

### Cichoriin reduces IL-1β and IL-18 release

**Fig. 2:**
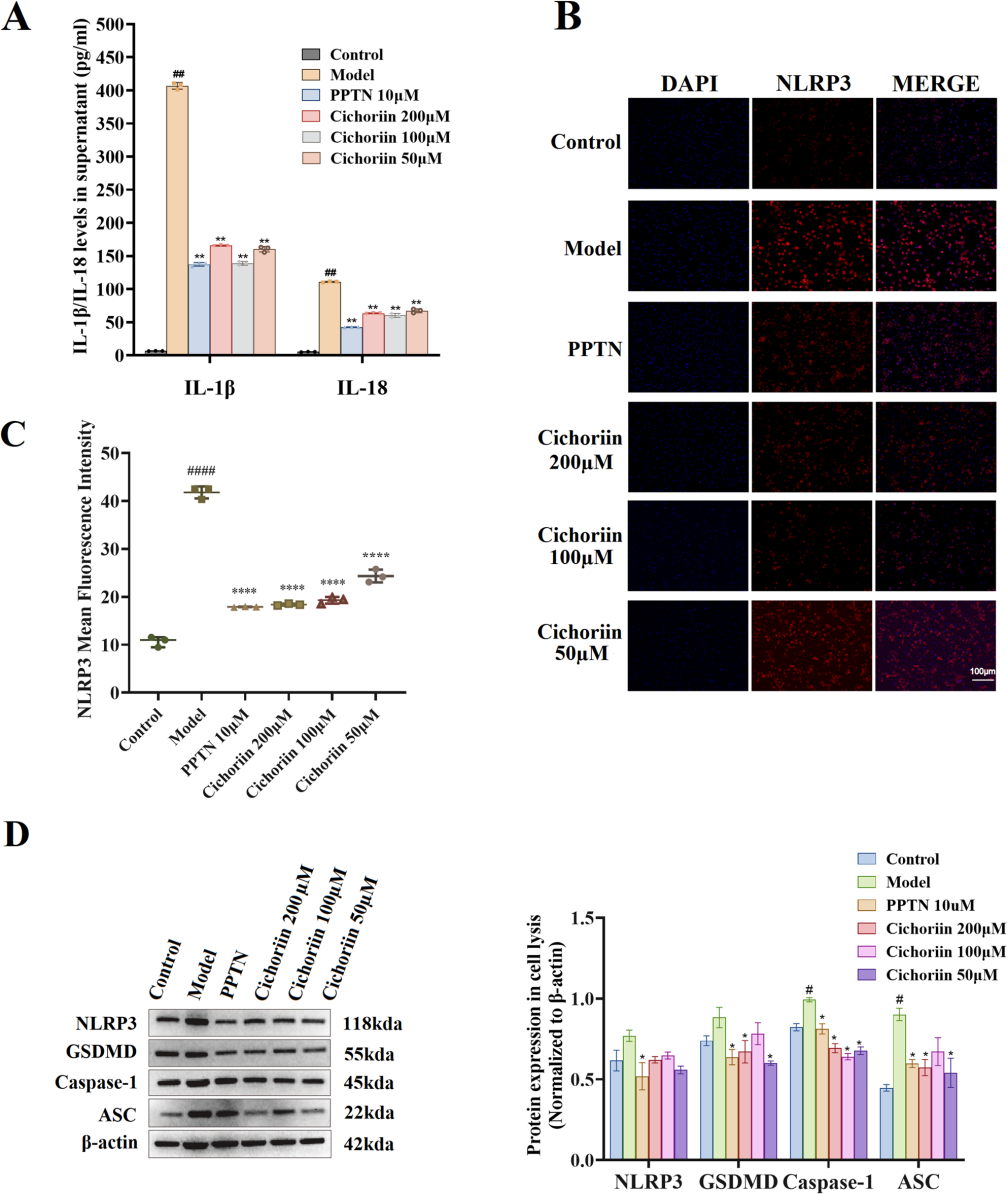
(**A**) Levels of IL-1β and IL-18 in cell supernatant; (**B**) Immunofluorescence image of NLRP3 expression in THP-1-derived macrophages; (**C**) Mean fluorescence intensity of NLRP3; (**D**) Western blot analysis of the expressions of NLRP3, GSDMD, caspase-1, and ASC. Values were expressed as means ± sd. # *P* < 0.05, ## *P* < 0.01 compared with blank control group; * *P* < 0.05, ** *P* < 0.01,***P < 0.001, ****P < 0.0001 compared with model group

MSU treatment markedly increased the levels of IL-1β and IL-18 in the culture supernatant compared with blank group (*P* < 0.01), as shown in Fig. 2A. Treatment with either PPTN or cichoriin significantly attenuated this elevation (*P* < 0.01). Although the PPTN group exhibited the lowest cytokine concentration, the differences between PPTN and any cichoriin doses were not statistically significant (*P* > 0.05), indicating comparable anti-inflammatory efficacy. Among the three cichoriin doses, the medium concentration produced the greatest inhibition, and no clear dose-dependency was observed. Collectively, these results demonstrated that cichoriin effectively suppressed MSU-induced IL-1β and IL-18 production.

### NLRP3 immunofluorescence staining

Representative micrographs were shown in Fig. 2B, and corresponding relative integrated optical density (IOD) values were plotted in Fig. 2C. In resting macrophages, NLRP3 is a cytoplasm protein and emits weak red fluorescence. MSU stimulation strongly intensified this signal, indicating robust up-regulation of NLRP3. Treatment with PPTN or any dose of cichoriin markedly attenuated the fluorescence intensity, demonstrating effective suppression of NLRP3 expression. Quantitative analysis revealed that the relative IOD of NLRP3 in MSU group was significantly higher than that in blank control group (*P* < 0.0001). All compound-treated groups exhibited a pronounced reduction in NLRP3 signal compared with MSU group (*P* < 0.0001), confirming that both PPTN and cichoriin can interfere with NLRP3 inflammasome activation.

### Cichoriin down-regulates the expressions of NLRP3, caspase-1, GSDMD, and ASC

In order to further explore whether cichoriin acts by suppressing the activation of NLRP3 inflammasome, the expressions of key pyroptosis-related proteins (NLRP3, caspase-1, GSDMD, and ASC) were detected by using western blot assay (Fig. 2D). The expressions of these proteins were up-regulated in MSU group relative to blank control group. Compared with MSU group, PPTN and cichoriin groups could significantly reduce the expression of NLRP3, caspase-1, GSDMD, ASC (*P* < 0.05). Compared with PPTN group, the high, medium, and low doses of cichoriin gave comparable band intensities for NLRP3, caspase-1, GSDMD and ASC (*P* > 0.05). These data indicate that cichoriin has the potential to regulate NLRP3 inflammasome assembly.

### CETSA

**Fig. 3:**
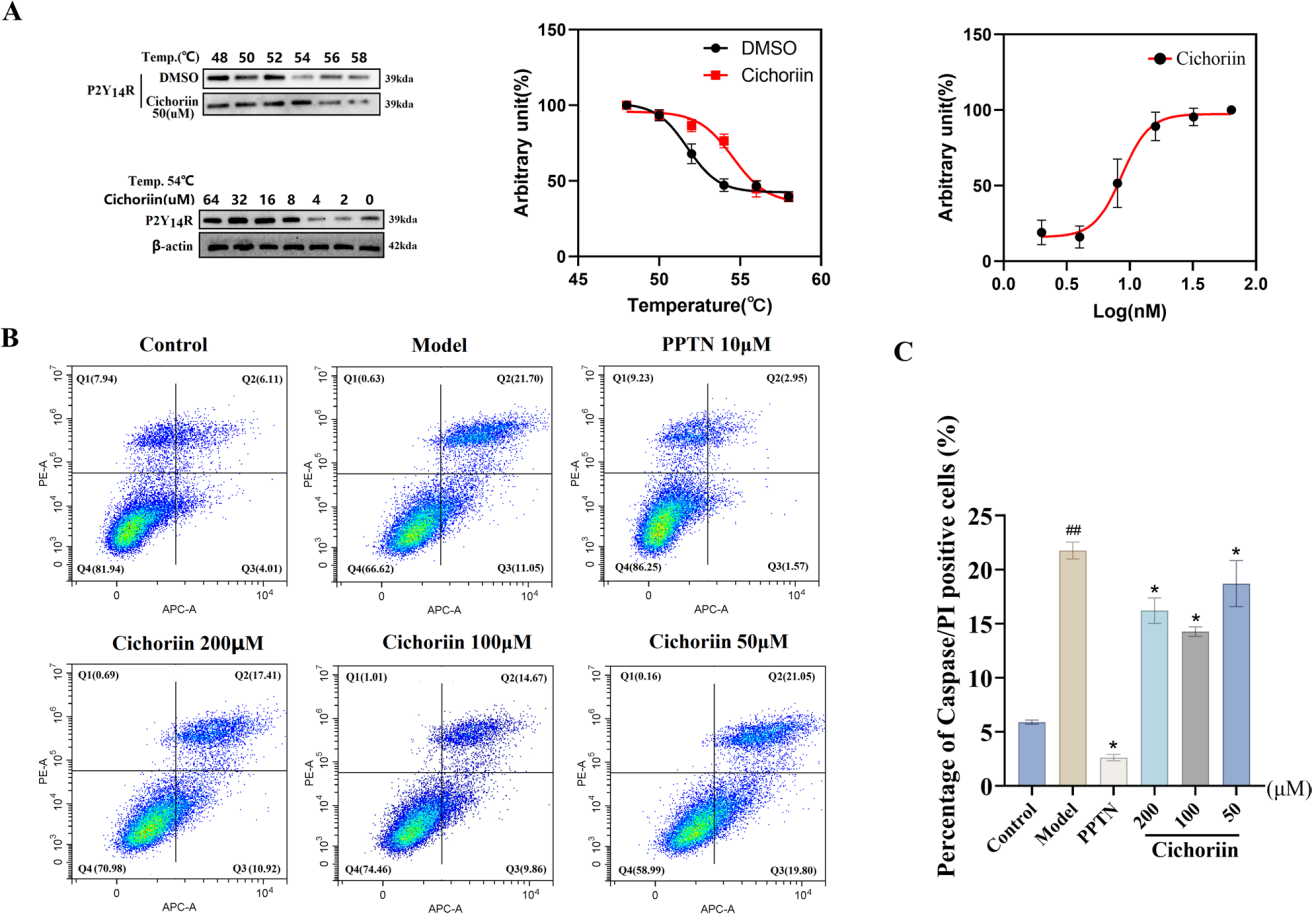
(**A**) Cellular thermal shift assay result between cichoriin and P2Y_14_R; (**B**) Flow cytometric analysis of caspase-1/PI-stained cells; (**C**) The proportion of caspase-1/PI double-positive cells. ## *P <* 0.01 compared with blank control group; * *P <* 0.05 compared with model group

In order to confirm the interaction between P2Y_14_R and cichoriin, CETSA was used to monitor the thermal stability of P2Y_14_R at 50 µM of cichoriin. As shown in Fig. 3A, cichoriin could significantly enhance the thermal stability of P2Y_14_R. At 54℃, the thermodynamic stability of P2Y_14_R exhibited a concentration-dependent manner in the presence of cichoriin. These results suggested that cichoriin can bind tightly to the active pocket of P2Y_14_R.

### Pyroptosis analysis

Flow-cytometry data were acquired with CytExpert software, and were presented in Fig. 3B. The percentage of caspase-1/PI double-positive cells for each group was plotted in Fig. 3C. Compared with blank control group, MSU stimulation markedly increased the proportion of double-positive cells (*P* < 0.01). Treatment with high, medium, or low dose of cichoriin, as well as with PPTN, significantly reduced this proportion compared with MSU group (*P* < 0.05). The attenuated pyroptosis rate mirrors the decline in MSU-driven inflammation. These findings indicated that cichoriin could effectively suppress pyroptosis and regulate MSU-driven inflammation.

### Homology modelling and qualitative evaluation of P2Y_14_R

**Fig. 4:**
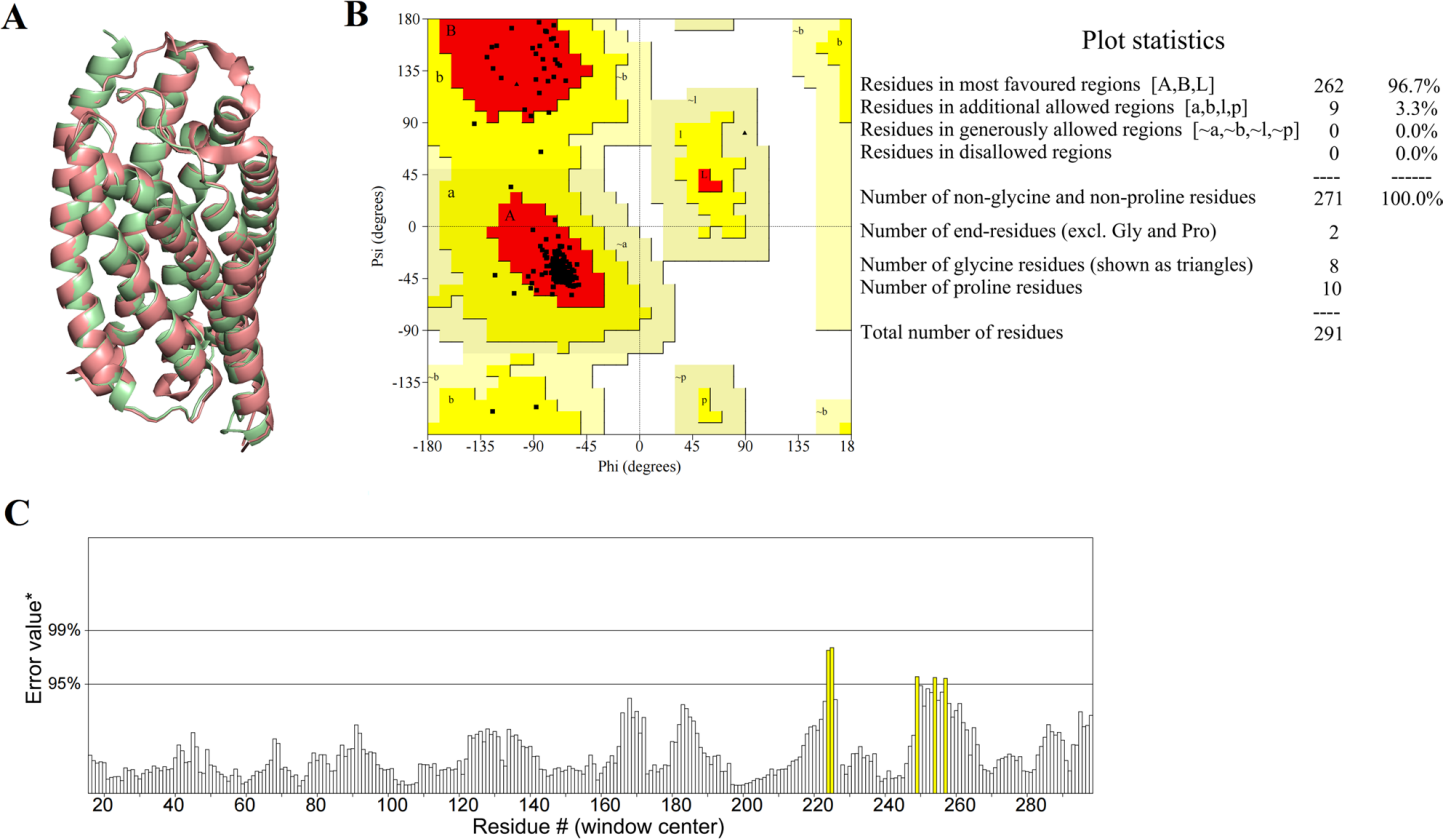
Quality assessment of P2Y_14_R model-structure. (**A**) The superimposition of P2Y_14_R homology modelling structure onto template structure; (**B**) Ramachandran plot of model structure; (**C**) ERRAT plot of homology model

The three dimensional (3D) structure of P2Y_14_R was constructed by homology modelling method followed by evaluation via PROCHECK, ERRAT, Molprobity, and QMEANDisco tools. The superimposition of P2Y_14_R homology modelling structure onto template structure was shown in Fig. 4A. The modeled 3D structure of P2Y_14_R was assessed by PROCHECK through ramachandran plot calculation. The ramachandran plot analysis showed 96.7% of residues in most favoured regions and 3.3% of residues in additional allowed regions (Fig. 4B), indicating that the quality of the modeled structure was much higher. ERRAT obtained from SAVES server could examine non-bonded atom-atom interactions and determine the structure’s reliability when assessing the amino acid environment. The overall estimated quality factor of ERRAT was 98.221, suggesting that the structure is of good quality and better reliability, as shown in Fig. 4C. Molprobity and QMEANDisco were used for further evaluation of the modeled structure quality. The structure displayed a molprobity score of 0.88, indicating the acceptable quality of this model. A QMEANDisco score of 0.66 further validate the structural reliability for this modeled structure. All these validation parameters disclosed the confidence over the modeled structure.

### MD simulation studies

The current study aimed to evaluate the interactions between cichoriin and P2Y_14_R. In order to obtain the binding structure of protein-ligand, molecular docking method was applied, and the complex conformation with the lowest binding energy was chosen for the following molecular dynamics simulation study. A 100 ns MD simulation of an apo form of P2Y_14_R as well as P2Y_14_R-PPTN and P2Y_14_R-cichoriin complexes was conducted to explore the complexity conformation and acquire conformational flexibility and stability of protein-ligand complex, and the overall MD simulation results were interpreted in RMSD, RMSF, Rg, SASA, hydrogen bond interactions, DCCM, FEL, and MM-PBSA.

**Fig. 5:**
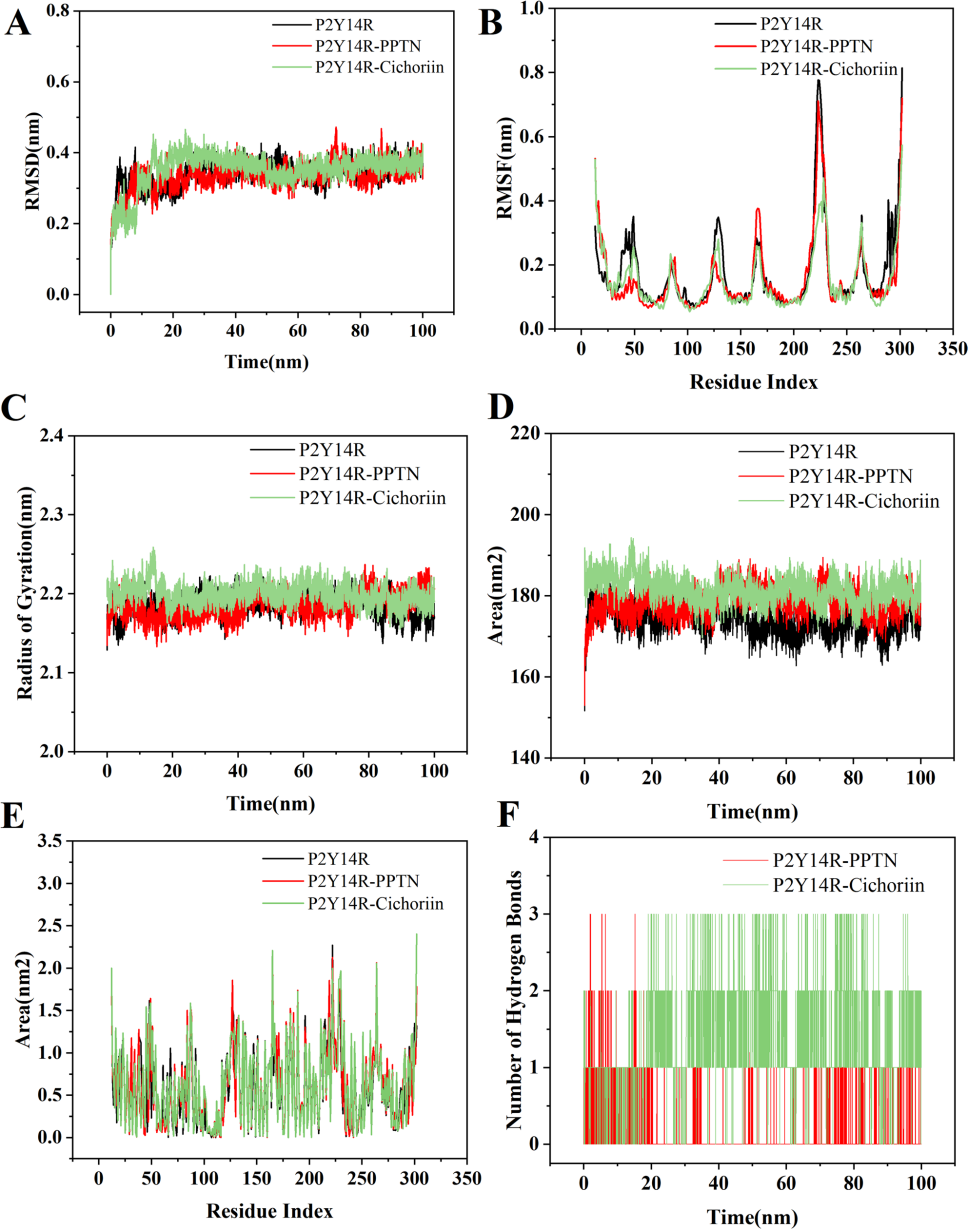
(**A**) RMSD values for the backbone atoms of apo form of P2Y_14_R, P2Y_14_R-PPTN, and cichoriin-P2Y_14_R complexes. (**B**) RMSF values for the Cα atoms of apo form of P2Y_14_R, P2Y_14_R-PPTN, and cichoriin-P2Y_14_R complexes. (**C**) Rg profile of apo form of P2Y_14_R, P2Y_14_R-PPTN, and cichoriin-P2Y_14_R complexes. (**D**) SASA for each residues of P2Y_14_R. (**E**) SASA in nm^2^ for P2Y_14_R system over 100 ns MD simulation. (**F**) The number of hydrogen bonds for PPTN and cichoriin with P2Y_14_R

RMSD represents the protein complex stability. It is benefit to assess the equilibration period for the MD run and monitor the the dynamic changes for protein and ligand at a particular condition throughout the MD time. In the current study, RMSD analysis showed the P2Y_14_R RMSD fit with PPTN and cichoriin RMSD over a 100 ns time scale (Fig. 5A). The backbone RMSD of protein varied from about 0.3 nm to 0.4 nm, and had an average value of 0.35 nm. The cichoriin-P2Y_14_R complex reached equilibrium after about 30 ns MD simulations with lower RMSD fluctuation. RMSF denotes the fluctuation of each residues and quantifies the localized variations along protein chain. The peaks on RMSF plot represent the highest fluctuation region in protein throughout the MD simulation. The RMSF profiles were calculated by residues index Cα of protein during the MD simulation, as illustrated in Fig. 5B. The N- and C-terminal tails as well as loop regions exhibited greater fluctuations compared with other regions of P2Y_14_R protein. The RMSF value was decreased when P2Y_14_R was bound to cichoriin. The decreased fluctuations were related to the cichoriin-binding cite. The Rg measures are benefit to understanding the compactness, molecular stability, and folding of protein structure. The Rg value of the complex was between about 2.14 and 2.24 nm over the 100 ns simulation (Fig. 5C), indicating a stable and compactness complex. SASA represents a geometric measure of the part of a protein that would be surrounded by solvent molecules, and helps to understand the extend of protein surface exposed to surrounding solvent molecules. As shown in Fig. 5D, the SASA values of P2Y_14_R were calculated, and the volume against the time was plotted. SASA values ranged from approximately 170 nm^2^ to 190 nm^2^. The SASA per residue was calculated, as shown in Fig. 5E, which could provide the information about the residues exposed to the solvent molecules. The volume of PPTN and cichoriin systems stayed similar, and slightly increased volume after binding to ligand was observed. As shown in Fig. 5B and E, the residues with lesser RMSF value generally had lower SASA value. The residues with higher SASA were mainly located at terminal parts and loop regions. In the active site of P2Y_14_R, the small increased SASA indicated the accessibility to solvent after binding to cichoriin. Subsequently, an intermolecular hydrogen bond analysis was conducted for PPTN and cichoriin with P2Y_14_R, and was benefit to understand protein-ligand interactions. As shown in Fig. 5F, a higher number of hydrogen bonds was formed in the complex of cichoriin-P2Y_14_R during the period from 20 to 100 ns, and the maximum hydrogen bonds formed were 3. The presence of intermolecular hydrogen bonds in the system during the MD simulations further indicates the stability for the complex.

**Fig. 6:**
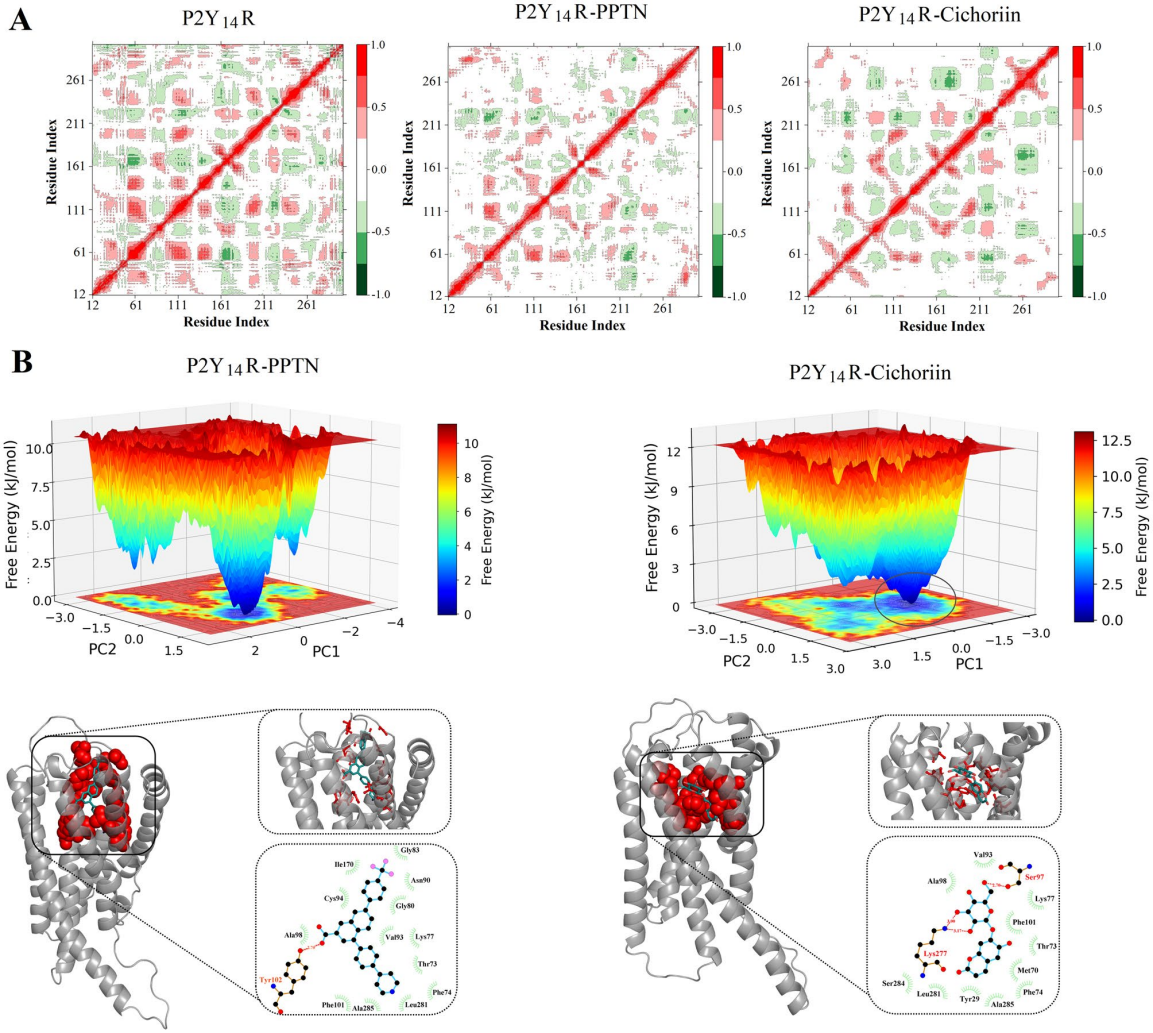
(**A**) Dynamic cross-correlation map of Cα atoms of apo form of P2Y_14_R, P2Y_14_R-PPTN, and cichoriin-P2Y_14_R. (**B**) The FEL of P2Y_14_R-PPTN and P2Y_14_R-cichoriin depicted as a function of PC1 and PC2. The lower panel represents the structure with minimal energy

DCCM is crucial for understanding the correlated motions between residues and examining the important interaction networks. DCCM analyses were performed for elucidating the impact of PPTN and cichoriin on the internal dynamics of P2Y_14_R. The correlation values ranged from − 1 to 1. As shown in Fig. 6A, the apo form of P2Y_14_R exhibited extensive positive and negative correlations between residues, highlighting a high-level of internal motion and flexibility. Upon binding with PPTN and cichoriin, the overall correlations were sharply decreased. In the complex of P2Y_14_R-cichoriin, the strongly altered correlations were observed in the region of residues 200–303.

FEL plot denotes the thermodynamics and kinetics for the complex of protein and ligand through the simulation. The contour 3D FEL plot of cichoriin-P2Y_14_R complex was generated in Fig. 6B for identifying the energy minima and the corresponding representative lowest-energy conformation. Principal component analysis could assess the overall expansion for a protein throughout MD simulation, and reveal the underlying atomic fluctuations for the structure. The first two principal components (PC1 and PC2) were used to map clusters onto FEL for identifying stable conformations. Within the 3D FEL image, the color spectrum was ranged from conformational states with lowest energy (blue) to with highest energy (red), indicating the different range of Gibbs energies. A distinct global minimum energy cluster marked with deeper blue had a relatively concentrated distribution, indicating a good stability of complexes. As seen by the dark blue color in the FEL plot, the complexes of P2Y_14_R-PPTN as well as cichoriin-P2Y_14_R showed stable conformation at free energy at lower than 2 kJ/mol. The least energy conformation states for P2Y_14_R-PPTN and P2Y_14_R-cichoriin complexes were extracted from FEL analysis, as shown in Fig. 6B.

**Fig. 7:**
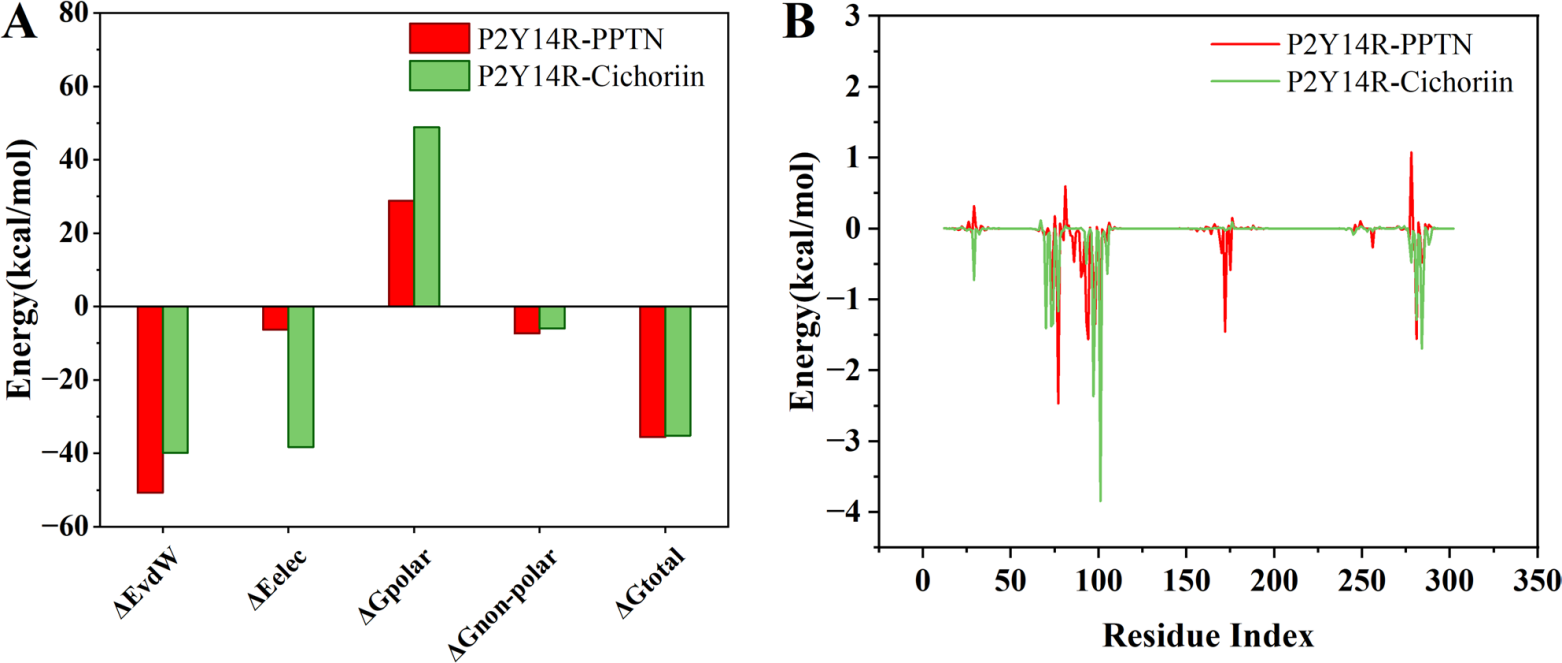
(**A**) Representative contributions of energy components for binding free energy for P2Y_14_R-PPTN and cichoriin-P2Y_14_R. (**B**) Binding free energy decomposition for the key residues in P2Y_14_R when bound by PPTN and cichoriin

In MD simulation, the calculation of binding free energy plays a vital role in determining interaction between ligand and protein. The MM-PBSA method was applied to calculate the free interaction energy for the complexes of P2Y_14_R-PPTN and P2Y_14_R-cichoriin in order to evaluate the binding affinity on the poses with lowest local free energy. The binding energy assessment was conducted via gmx_MMPBSA tool. The binding free energy was calculated by using the snapshots obtained from the most stable states in FEL plot. The energetic parameters contributing to the binding free energy for the two complexes were shown in Fig. 7A. The calculated binding free energy is -35.13 kcal/mol, indicating a strong interaction between cichoriin and P2Y_14_R. As seen from the binding free energy components, van der Waals interactions ( E_vdW_ = -39.83 kcal/mol ) and solvation nonpolar contributions ( G_non−polar_ =−5.94 kcal/mol) were benefit to the binding of cichoriin to P2Y_14_R. The electrostatic component in the binding energy also was a crucial contributor in the binding of cichoriin to P2Y_14_R. The polar part of solvation free energy ( G_polar_ = 48.87 kcal/mol ) is an unfavorable contribution to the binding affinity of this complex for ligand-binding pocket exposed to the solvent. The binding free energy decomposition was conducted in order to characterize and identify the detailed effects of individual residues to the intermolecular interaction energies, as shown in Fig. 7B. Based on the estimated binding free energy, the favorable and unfavorable interactions were obtained. The highly-favorable energy contributions were included amino acids Tyr29, Met70, Thr73, Phe74, Phe76, Lys77, Ile78, Val93, Ser97, Ala98, Phe101, Asn104, Met105, Lys277, Glu278, Leu281, Leu283, Ser284, Ala285, Val288, Vys289. Amino acids Asp67, Asp81, Tyr106, Lys176, and Arg274 were not favorable to the interaction between cichoriin and P2Y_14_R.

## Discussion

Gout is an autoinflammatory disorder triggered by the deposition of MSU crystals. The excessive activation of NLRP3 inflammasome could be triggered by MSU crystals [29]. The assembly of NLRP3 inflammasome is composed of sensor NLRP3, adaptor ASC, and effector pro-caspase-1 [30]. The activation of caspase-1 further promotes pro-IL-1β maturation, which leads to the secretion of inflammatory cytokine IL-1β [31]. P2Y_14_R, a G protein-coupled receptor, has been found to be expressed in a variety of tissues, especially in immune cells, and its activation plays a crucial role in regulating immune responses and inflammatory processes [32, 33]. The activation of P2Y_14_R could suppress intracellular cAMP production and further modulate downstream signaling cascades [34, 35]. It has been found that P2Y_14_R could accelerate macrophage pyroptosis through cAMP-dependent activation of NLRP3 [20]. The inhibition of P2Y_14_R activity could block the activation of NLRP3 inflammasome and restrain MSU-induced pyroptosis [21]. Therefore, P2Y_14_R represents a potential target for the treatment of inflammatory diseases. Conventional therapeutic approaches for gout have limitations, such as adverse side effects, drug-drug interactions, and poor patient adherence [36]. In this context, there is a necessity to develop new anti-gout drug candidates. In this study, scientific validations were used to identify and authenticate the potential of cichoriin to treat inflammatory reaction in gout by using a rigorous and systematic approach beginning with the inhibition of P2Y_14_R. Cichoriin is a type of coumarin. A large number of coumarin derivatives have potential anti-inflammatory and antioxidant activities [37], show influences on purine metabolism, and their disorders lead to gout [38]. The cortex fraxini total coumarin could relieve pain for the patients with primary acute gouty arthritis [39]. These previous reports indicate the therapeutic effects of coumarin and their derivatives on gout. Cichoriin is the extract prepared from Podospermum canum (Asteraceae) [40], Cichorium Pumilum (chicory) [41], Fraxinus hupehensis [42], and callus tissue of C. intybus [43]. They exhibit anti-inflammatory, antioxidant, diuretic, anti-hyperuricemic and anti-tumor effects [44–46]. Cichoriin shows potential of anti-inflammatory and anti-microbial [47, 48]. It has been reported that the treatment with cichoriin could improve lipid profile and oxidative balance state for high fat diet-induced obese rates [49]. We observed that cichoriin could inhibit the activity of P2Y_14_R. In order to further evaluate the potential of cichoriin to treat gout, cell viability, ELISA, immunofluorescence staining, CETSA, and flow cytometry assays were performed. Our results showed that cichoriin could bind to P2Y_14_R, down-regulate the expressions of NLRP3, caspase−1, GSDMD, and ASC, increase IL−1β and IL−18 levels, and decrease the percentage of caspase−1/PI double-positive cells. These results indicated that cichoriin may attenuate gouty inflammation and pyroptosis in THP−1 macrophages by blocking P2Y_14_R/NLRP3/caspase−1 signaling axis. The findings were consistent with the results of previous studies on the anti-inflammatory effects of cichoriin.

In order to study the mechanism of the interactions between P2Y_14_R and cichoriin, in silico methods were applied to simulate biological interactions. These techniques can sharply reduce the need of extensive experimental testing and further reduce the time and resource costs.

The 3D structure of P2Y_14_R was obtained from homology modelling. After that, docking method could provide crucial insights into the interactions between ligand and protein. The binding behaviour between cichoriin and P2Y_14_R was further studied through MD simulation. In generally, MD trajectory could be used as a specific marker to monitor the trends of energy and molecular deformations. For parameters obtained from MD simulation, the average RMSD value of cichoriin-P2Y_14_R complex was stabilized after 30 ns, indicated that cichoriin imposed a significant interaction to P2Y_14_R. Rg could be used to describe the compactness of the system. The stable Rg values for the complex of P2Y_14_R and cichoriin indicated that the structure of this complex was remained compacted during the MD simulation. The stable SASA values for the complex of cichoriin and P2Y_14_R denote a stable binding interaction, and cichiion was well-encapsulated within the binding pocket of P2Y_14_R. The hydrogen bonds were recorded throughout the MD simulation, which are the main stabilizing interaction factor between cichoriin and P2Y_14_R during the whole simulation. These findings indicated that cichoriin could stabilize the structure of P2Y_14_R, and further potentially enhance its inhibitory ability. The decreased overall DCCM values demonstrated that cichoriin could reduce conformational flexibility and internal dynamics of P2Y_14_R and significantly stabilize P2Y_14_R. These results aligned with RMSD, RMSF, Rg, and SASA analyses, further supporting the potential of cichoriin as a potent P2Y_14_R inhibitor. FEL plot for cichoriin-P2Y_14_R complex was generated via the calculation of PC1 and PC2 as reaction coordinates for depicting the global minima energy conformation of this complex. A noticeable large global energy minima basin was observed in FEL plot, which suggested a strong and stable conformation of cichoriin-P2Y_14_R complex. MM-PBSA binding free energy calculations and per-residue decomposition analysis were further used to study the binding affinity of cichoriin towards P2Y_14_R. The negative free energy denotes the favorable reactions, and lower binding energy could enhance the interaction. Based on the results of MM-PBSA and residual binding energy decomposition analysis, it was conducted that the complex of cichoriin-P2Y_14_R was energetically stable. Amino acids Asp67, Asp81, Tyr106, Lys176, and Arg274 were not favorable to the interaction between cichoriin and P2Y_14_R, which may be related to their position in the active site cavity. Among the most favorable amino acid residues in MM-PBSA analysis, Tyr29, Met70, Thr73, Phe74, Lys77, Val93, Ala98, Phe101, Leu281, Ser284, Ala285 were involved in the hydrophobic contracts in the binding of cichoriin to P2Y_14_R. Furthermore, Ser97 and Lys277 interacted with cichoriin via hydrogen bonds. Among these amino acid residues, Lys 77 and Tyr277 were reported to involve in the binding of ligand to P2Y_14_R, especially Lys277 was observed as favorable residue in the binding pocket of P2Y_14_R [50]. The critical residues at binding site of P2Y_14_R had stable fluctuation with lower RMSF value over the whole 100 ns simulation. RMSF can be used as an indicator of amino acid residual mobility. High RMSF values denote a large degree of mobility for the receptor. Conversely, low RMSF values denote more stable and rigid regions. These results indicated the stable complex of cichoriin-P2Y_14_R.

In this study, the findings could provide scientific evidence to support the potential of cichoriin in treating MSU-induced gouty inflammation and offer a meaningful perspective on its mechanism of action. However, there were some limitations. First, multiple repetitions were required for MD simulation; Second, additional mice models are needed for further validation studies. Third, rescue experiments were needed to confirm the signaling mechanism. Forth, direct high-resolution morphological evidence—such as immunofluorescence imaging of cytoskeletal rearrangements or electron microscopy visualization of membrane pore formation—was not provided. Finally, a more comprehensive assessment of underlying molecular mechanism is still needed in the following study.

## Conclusion

In summary, findings of this study demonstrated that cichoriin may suppress MSU-induced pyroptosis in THP-1 macrophages and attenuate the associated inflammatory response by blocking the P2Y_14_R/cAMP/NLRP3 signaling axis. These results provided initial proof-of-concept for cichoriin as a potential therapeutic agent for MSU-induced gouty inflammation and warranted further validation in animal models. Elucidating the anti-inflammatory mechanisms of cichoriin not only deepens our understanding of their scientific basis but also maximizes its clinical utility, offering a promising new avenue for the treatment of gout and related inflammatory disorders.

## Supplementary Information

Below is the link to the electronic supplementary material.

Supplementary Material 1

## Data Availability

Data available on request from the authors.
