# Supplementary material for: Mechanistic insights into how cichoriin inhibits P2Y14R to suppress MSU-induced gouty inflammation
Source: BMC Biotechnol. 2026 Apr 15;26:69. doi: 10.1186/s12896-026-01151-z (PMC13196109; doi:10.1186/s12896-026-01151-z)
Supplement: Supplementary file 1 — Supplementary Material 1 [file 12896_2026_1151_MOESM1_ESM.docx]

****Original western blot image****


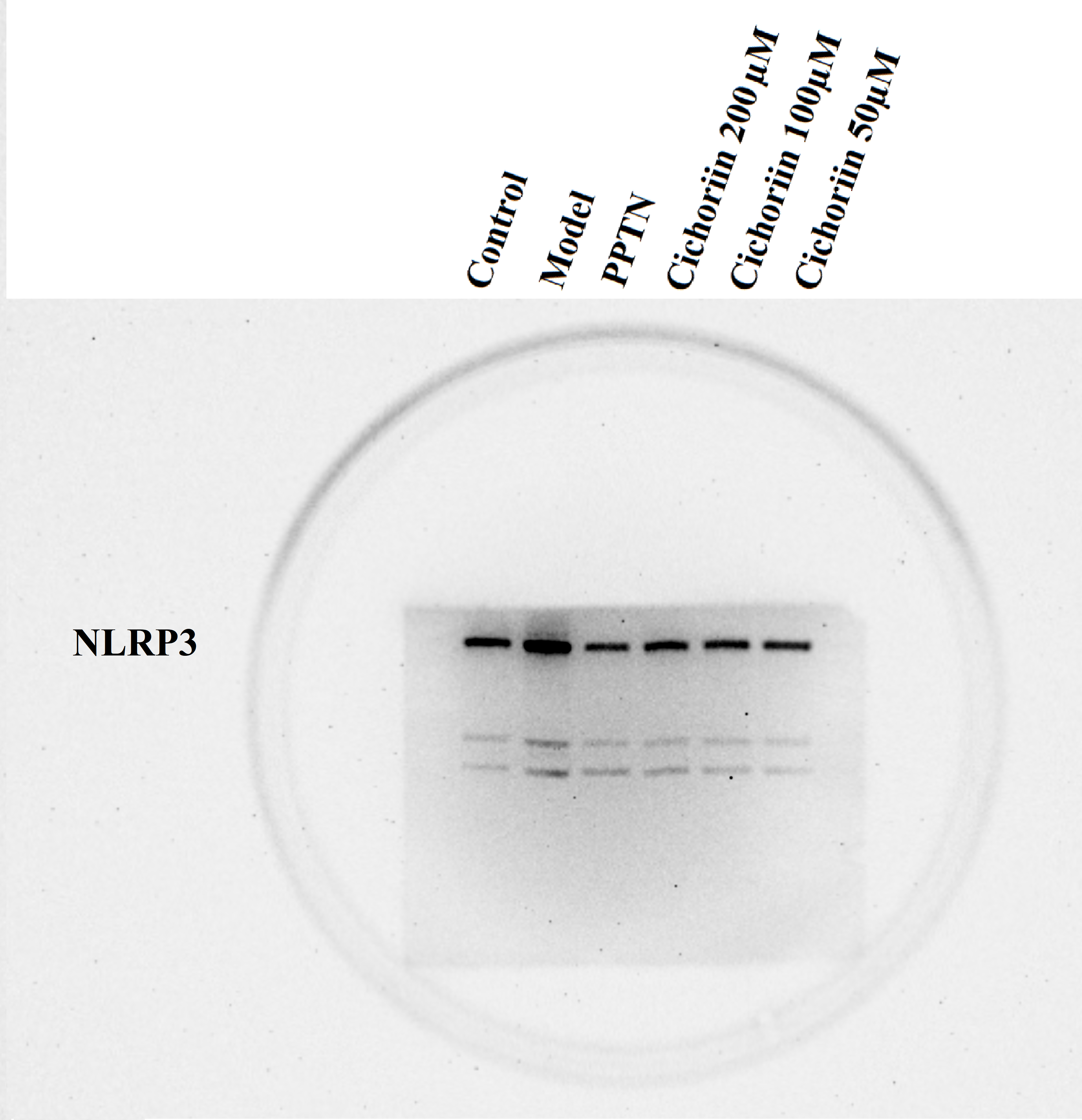


Figure 1 Western blot analysis of Nod-like receptor family, pyrin domain containing 3 (NLRP3) expression.


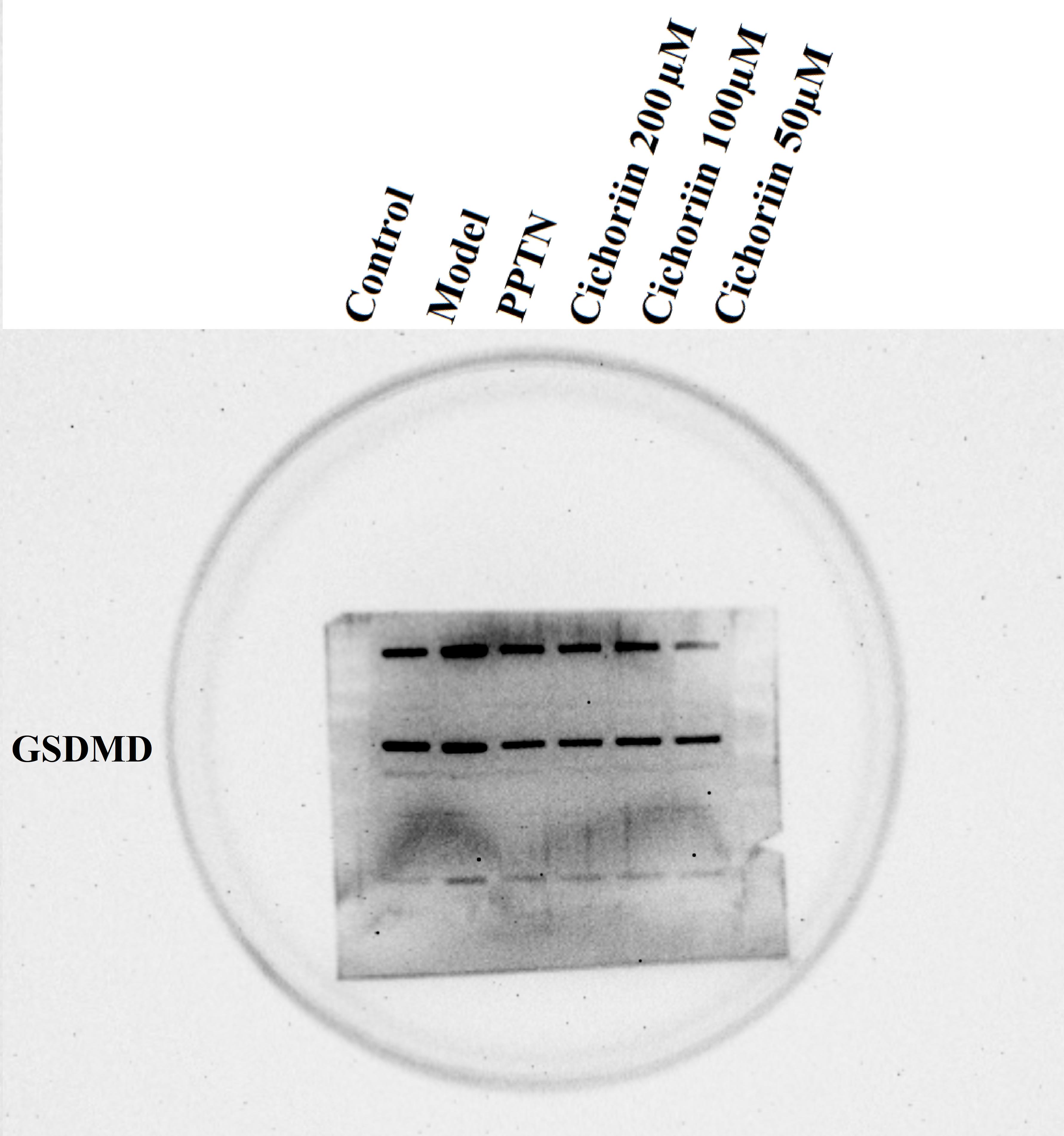


Figure 2 Western blot analysis of gasdermind D (GSDMD) expression.


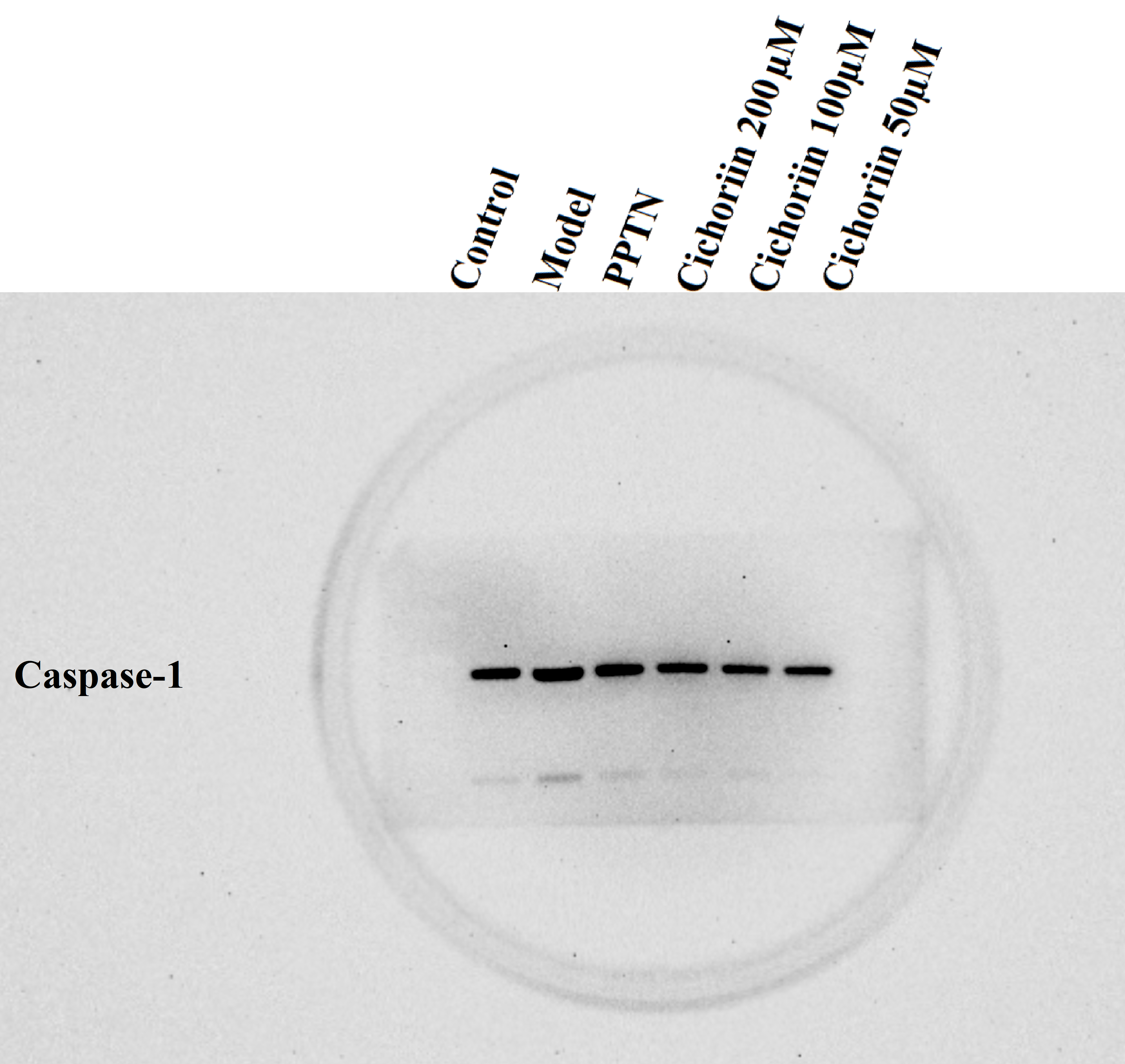


Figure 3 Western blot analysis of caspase-1 expression.


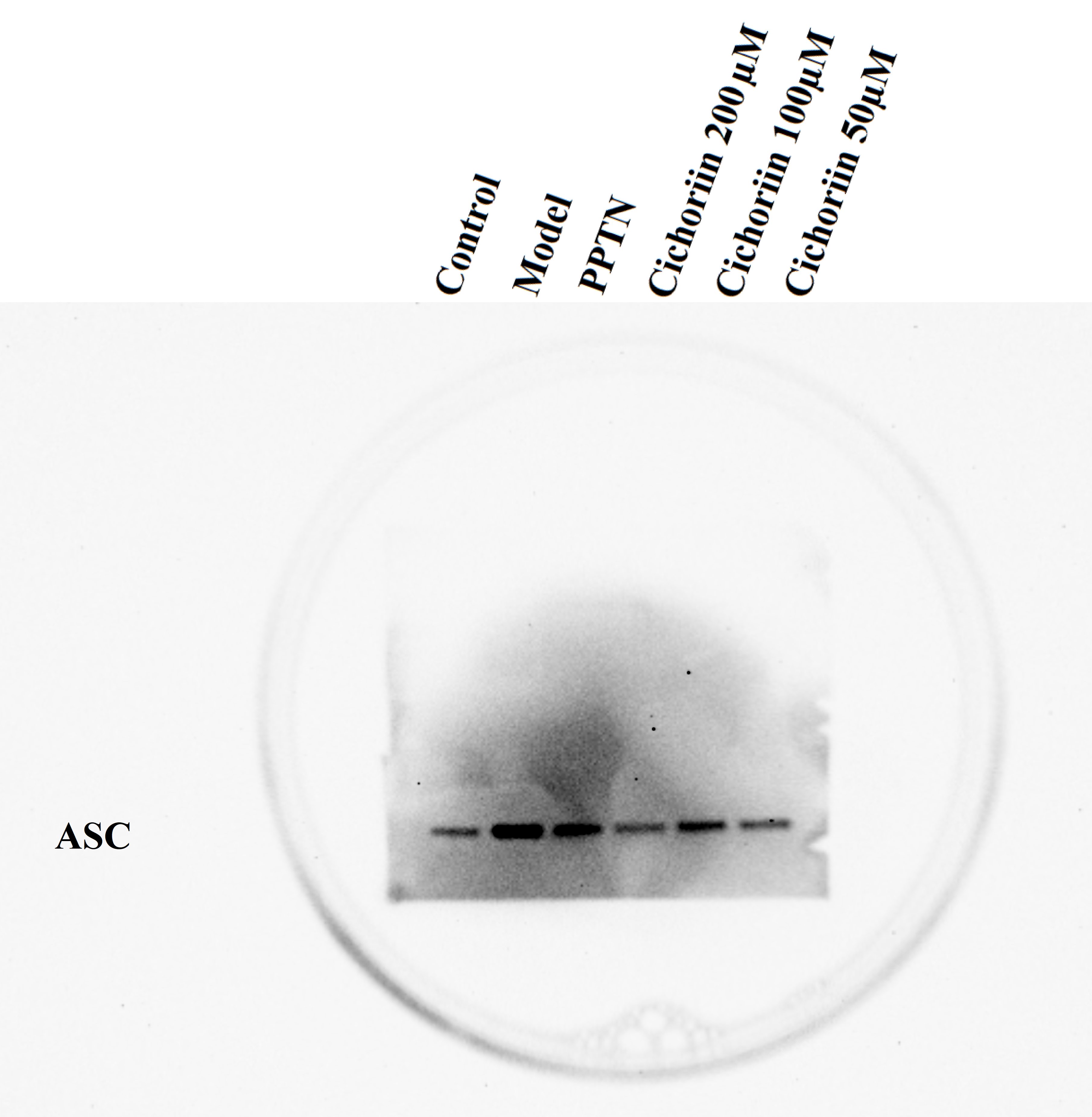


Figure 4 Western blot analysis of apoptosis-associated speck-like protein (ASC) expression.


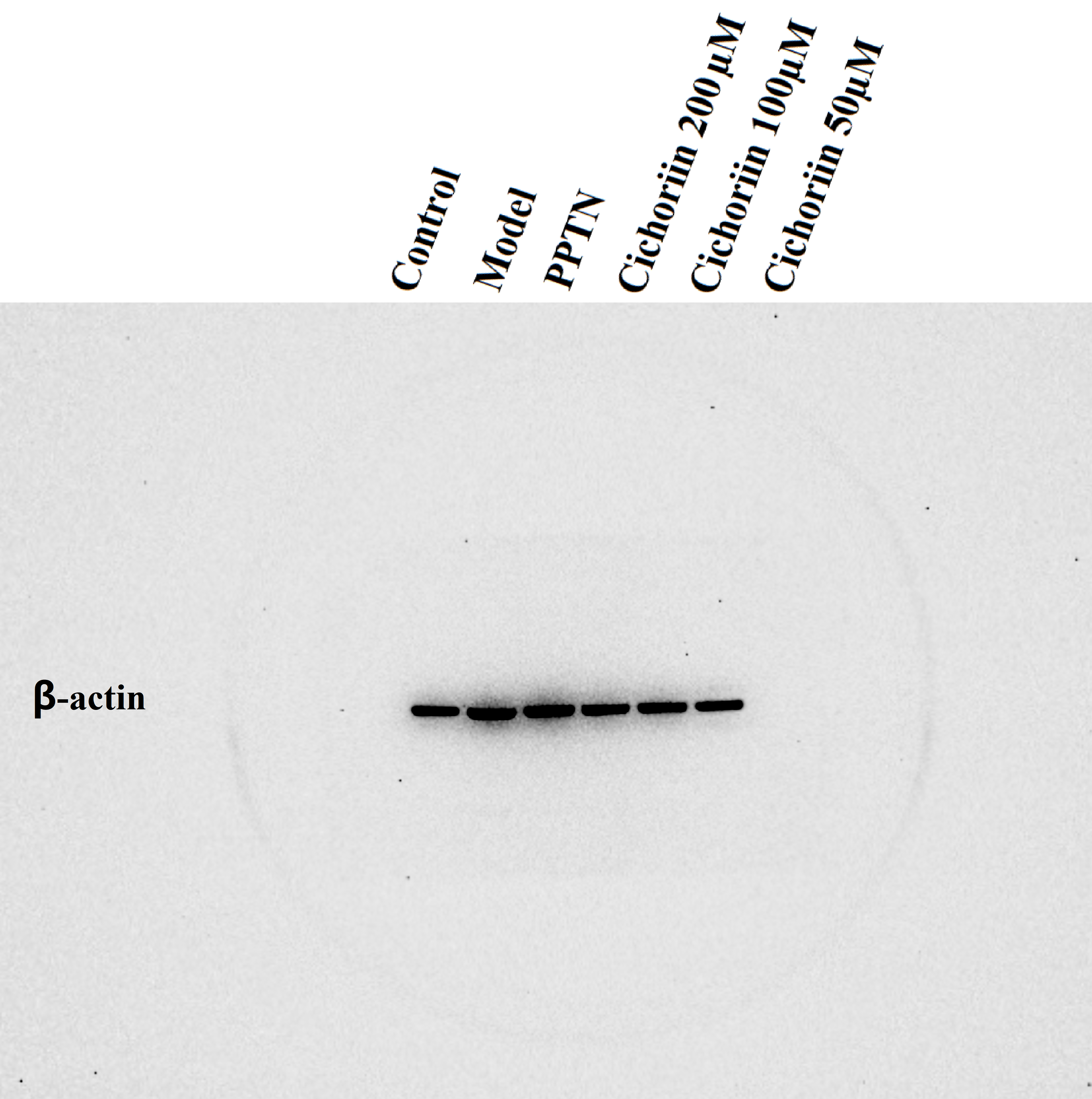


Figure 5 Western blot analysis of β-actin expression.
